# Predicting Antigen Presentation—What Could We Learn From a Million Peptides?

**DOI:** 10.3389/fimmu.2018.01716

**Published:** 2018-07-25

**Authors:** David Gfeller, Michal Bassani-Sternberg

**Affiliations:** ^1^Department of Oncology, Ludwig Institute for Cancer Research, University of Lausanne, Lausanne, Switzerland; ^2^Swiss Institute of Bioinformatics (SIB), Lausanne, Switzerland; ^3^Department of Oncology, Ludwig Institute for Cancer Research, University Hospital of Lausanne, Lausanne, Switzerland

**Keywords:** human leukocyte antigen peptidomics, human leukocyte antigen ligand prediction, antigen presentation, T cell epitope, computational immunology

## Abstract

Antigen presentation lies at the heart of immune recognition of infected or malignant cells. For this reason, important efforts have been made to predict which peptides are more likely to bind and be presented by the human leukocyte antigen (HLA) complex at the surface of cells. These predictions have become even more important with the advent of next-generation sequencing technologies that enable researchers and clinicians to rapidly determine the sequences of pathogens (and their multiple variants) or identify non-synonymous genetic alterations in cancer cells. Here, we review recent advances in predicting HLA binding and antigen presentation in human cells. We argue that the very large amount of high-quality mass spectrometry data of eluted (mainly self) HLA ligands generated in the last few years provides unprecedented opportunities to improve our ability to predict antigen presentation and learn new properties of HLA molecules, as demonstrated in many recent studies of naturally presented HLA-I ligands. Although major challenges still lie on the road toward the ultimate goal of predicting immunogenicity, these experimental and computational developments will facilitate screening of putative epitopes, which may eventually help decipher the rules governing T cell recognition.

## Introduction

Recognition of infected or malignant cells by T cells relies on the presentation of immunogenic self and non-self peptides at the cell surface. Two main pathways have been identified for antigen presentation and processing (1–3).

In the class I pathway, intracellular proteins are degraded into small peptides by the proteasome. These peptides are transported into the endoplasmic reticulum by the transporter associated with antigen processing (TAP) protein complex. There, they can bind to human leukocyte antigen class I (HLA-I) molecules in complex with beta2-microglobulin (β2m). After trafficking to the cell surface, the complexes may be recognized by CD8 T cells. HLA-I proteins are primarily encoded by three genes (HLA-A, HLA-B, and HLA-C), which are widely expressed in most cell types in human. In addition, specialized cell types can express HLA-E, HLA-F, or HLA-G genes. HLA-A, -B, and -C genes (hereafter referred to as HLA-I) are the most polymorphic genes in the human genome and over 12,000 distinct alleles are documented in the human population (4). Humans have in general different combinations of HLA-I alleles and, therefore, express up to six different HLA-I proteins (two for each gene). HLA-I molecules bind short peptides, mainly 9–11 amino acids, and different HLA-I alleles have distinct binding specificities, which implies that a broad spectrum of peptides can be displayed across different individuals.

In the class II pathway, peptides coming from the degradation of phagocytosed extracellular proteins are presented on HLA-II molecules for recognition by CD4 T cells (5). In addition, endogenous proteins can be presented on HLA-II molecules when degraded through autophagy (6). HLA-II proteins are encoded by several genes (HLA-DRA, HLA-DRB1,3,4,5, HLA-DPA1, HLA-DPB1, HLA-DQA1, HLA-DQB1) and also show a very high level of polymorphism in the humans (except for HLA-DRA). HLA-II form heterodimers (HLA-DRA/HLA-DRB1,3,4,5; HLA-DPA1/HLA-DPB1 and HLA-DQA1/HLA-DQB1). These dimers bind longer peptides (12–20 amino acids) within an open-ended peptide-binding site. Several other steps are involved in presentation of class II epitopes, such as loading on HLA-II molecules catalyzed by HLA-DM, peptide exchange catalyzed by HLA-DO, the presence of other enzymes such as cathepsins or pH gradients (7–10). Unlike HLA-I, HLA-II molecules are mainly expressed on specific professional antigen-presenting cells (pAPCs) such as dendritic cells or B cells (1), and rarely also by cancer cells such as melanoma (11). pAPCs can also uptake exogenous antigens and present them on HLA-I (12). This process is called cross-presentation, and it is crucial for priming of naïve T cells (13, 14). Altogether, the cellular antigen processing and presentation machinery ensures that the restrictive loading of either intracellular (class I) or extracellular (class II) peptides of the right length will take place in specialized cellular compartments.

The set of peptides presented on HLA molecules is called the HLA peptidome, also referred to as immunopeptidome or HLA ligandome. The HLA peptidome is a rich and complex repertoire of peptides that inform T cells about abnormalities in the genome, transcriptome, and proteome of infected or malignant cells (15–17). It is constantly modulated by HLA or peptides’ source protein expression levels, by posttranslational modifications and by the many enzymes, chaperones, and transporters that comprise the antigen processing and presentation machinery (7, 18–20). In particular, the catalytic subunits of the constitutive proteasome, the immunoproteasome, and the thymic proteasome are tightly regulated, leading to the production of distinct repertoires of presented peptides in different cell types and under different conditions (21–24).

Historically, the study and predictions of class I and class II T cell epitopes have mainly developed in the field of infectious diseases, and large datasets of peptides displayed at the surface of infected cells and recognized by T cells are available from HIV, dengue, or influenza (25, 26). In the field of cancer immunology, tumor-associated antigens (defined here as genes expressed in cancer cells and not, or very poorly, in normal cells) have received much attention for almost 30 years (27). For instance, T cell recognizing specific epitopes of NY-ESO or MAGE-1 proteins can be found in melanoma patients, indicating that the immune system can mount a response against tumor-specific antigens (27–29). More recently, many evidences have been accumulated indicating that cancer cells express unique mutated antigens, the so-called neoantigens, which can be recognized by the patients’ own (autologous) T cells (15, 30–35). The total number of somatic mutations in some tumors has been shown to correlate with the therapeutic efficacy of checkpoint blockade antibodies (36–39), suggesting that neoantigens could play an important role in tumor immune recognition. Moreover, several studies demonstrated clinical benefit mediated by the administration of highly enriched populations of neoantigen-reactive CD4^+^ and CD8^+^ T cells (34, 40) and by neoantigen-based vaccines (41, 42). Potential neoantigens are typically predicted first by identifying non-synonymous alterations from next generation sequencing data and second by predicting the binding to HLA molecules of peptides encompassing these non-synonymous genetic alterations (43). For these reasons, predictions of peptides presented on HLA-I and HLA-II molecules have gained renewed interest in the field of tumor immunology. Predicted neoantigens need to be then experimentally validated for HLA binding and immune recognition *in vitro* (44–47).

Here, we review approaches developed for predicting antigen presentation in human cells, with a focus on the latest experimental and computational developments to take advantage of in-depth and accurate mass spectrometry (MS) data of HLA peptidomics. Our aim is to describe the main steps of antigen presentation that proved to be successful in making quantitative predictions of antigens. The more biological aspects of antigen presentation and processing are covered in many other reviews (1–3, 8).

## Main Sources of HLA Ligand Data

A cornerstone in our ability to understand and predict antigen presentation has been the experimental identification of specific peptides interacting with HLA molecules. First, from an experimental point of view, HLA-I molecules do not fold stably in the absence of a ligand and, therefore, all biochemical, structural, and functional studies of HLA-I molecules rely on the availability of known HLA-I ligands. Second, all computational methods to predict HLA ligands at a large-scale use data-driven approaches based on sequence patterns identified within known ligands.

Two main classes of experimental assays have been developed to identify HLA ligands. The first class of assays consists of *in vitro* assays. For HLA-I molecules, refolding assays use conformational pan HLA-I antibodies to test whether the HLA-I complex is properly folded in the presence of a peptide (48–52). Peptide-rescuing assays consist of a photo-cleavable peptide that is stripped by UV radiation in the presence of another peptide (53–55). Competitive assays with radiolabeled peptides have been used to determine relative affinity (IC50) (56). Dissociation assays based on radiolabeled β2m have been used to probe the stability of peptide–HLA-I complexes (57, 58). Surface plasmon resonance techniques can be used to measure actual Kd values (59). *In vitro* binding assays have also been used for HLA-II ligands (60–62). Compared to class I ligands, screening of class II ligands at high throughput is facilitated since HLA-II molecules have an open-ended peptide-binding site. Therefore, peptides can be fixed on plates, which allow for the use of peptide microarrays (63), or directly encoded in different display systems such as phage or yeast display (64, 65).

*In vitro* binding assays play a central role in our ability to identify T cell epitopes from viral or cancer-specific antigens (66, 67). When used in combination with state-of-the art predictions tools, they enable rapid validation of predicted targets and are currently key to most neoantigen discovery approaches in cancer immunotherapy (30, 31, 68, 69). The main caveat of *in vitro* assays for HLA-I ligands is that the peptides have to be determined *a priori* and chemically synthesized, since both the C- and N-terminus of most HLA-I ligands need to be free in most cases. This limits the use of high-throughput and unbiased peptide screening technologies. Furthermore, the involvement of the components of the antigen-loading complex is missing in *in vitro* binding assays and, therefore, signals related to antigen loading *in vivo* cannot be captured.

The second type of experimental assays for HLA ligand identification is based on MS measurement of eluted HLA-binding peptides. This approach is the only methodology to comprehensively interrogate the repertoire of HLA ligands presented naturally *in vivo* (16, 18, 70, 71). The best-established HLA peptidomics methodology is based on immunoaffinity purification (IP) of HLA complexes from detergent solubilized lysates, followed by extraction and purification of the peptides. Typically, either anti-pan-HLA class I, anti-HLA-DR, or anti-pan-HLA class II monoclonal antibodies are used. The extracted peptides are then separated by high-pressure liquid chromatography and directly injected into a mass spectrometer. The resulting spectra obtained from the fragmentation of the peptides are compared with *in silico* generated spectra of peptides from protein sequence databases with MS search tools. Therefore, this search is limited to the available databases, usually the annotated human proteome. Moreover, peptides that have features that make them incompatible with ionization, those that are too hydrophobic or too hydrophilic, might not be detected with standards methods. With the new generation of mass spectrometers, thousands of HLA ligands can be identified per sample (15, 18, 72, 73). Cell lines, including human cancer cell lines, tumors, healthy tissues, and body fluids such as plasma have been subjected to HLA peptidomics analyses (18, 70–84). However, MS-based HLA peptidomics approaches have limited sensitivity and require a relatively large amount of biological sample (~1 cm^3^ of tissue or 1 × 10^8^ cells) (21). Furthermore, despite major improvement in the quality of HLA peptidomics data, one can never exclude small residual contaminations from co-eluted peptides or wrong annotation of spectra depending on the false discovery rate threshold used in spectral searches.

Dedicated proteogenomics computational pipelines for customized reference databases have been developed to expand the search space beyond the canonical human proteome. Customizing references to include somatic alterations observed in tumors have been used for direct identification of neoantigens by MS in murine and human cancer cell line models (31, 35, 80, 85), B cell lymphomas (86), and melanoma tissues (15). Similar approaches were also used for other cryptic peptides resulting from unconventional coding sequences in the genome (87) and new open reading frames (88) (see Non-Canonical HLA-I Ligands).

Historically, the first HLA-I motifs (e.g., HLA-A02:01) were found by looking at peptide sequences of eluted ligands identified by MS (89, 90). To overcome the fact that eluted peptides come from up to six HLA-I alleles in unmodified cell lines or tissue samples, two experimental approaches have been developed. The first approach consists of transfecting a soluble HLA allele into a cell line and pulling down only the soluble HLA-I molecules in complex with their ligands (91, 92). While it has been shown that the repertoire of peptides presented on transfected soluble HLA-I and the endogenous membranal HLA-I molecules are highly similar (93), the non-physiological expression level of the soluble HLA-I molecules and the potential different environment in the loading compartment could affect the overall peptide repertoire. Furthermore, endogenous HLA-I alleles can be shaded or naturally secreted from cells in culture (94) and could contaminate the secreted peptidome (75). Nevertheless, this approach proved very powerful to identify HLA-I motifs (77, 78, 95–97). Of particular interest is the study by Di Marco and co-authors where the motifs of 15 HLA-C alleles could be determined, together with motif for HLA-G01:01 (75). This detailed view of HLA-C alleles binding specificities enabled the authors of this study to identify for the first time specificity determinant residues in the HLA-C-binding site that provide likely molecular mechanisms explaining the differences observed between HLA-C binding motifs. The second experimental approach consists of using genetically modified cell lines that express only one allele (98, 99) and was used to study binding motifs of highly similar alleles, like HLA-B27:02 to HLA-B27:09 (100). This approach was also recently used to screen 16 HLA-A and HLA-B alleles, and this work confirmed that predictors trained on MS data could improve predictions of naturally presented HLA-I ligands (70). One advantage of this approach is that theoretically all peptides come from one single allele (see above for potential sources of contaminations). In parallel, we and others introduced computational techniques based on motif deconvolution (72, 101) and peptide clustering (102, 103) to accurately determine HLA-I restriction of eluted ligands from pooled samples without requiring to experimentally isolate each HLA-I allele and without relying on HLA-I ligand predictors (see below for a detailed description of these approaches).

### Comparison of MS and *In Vitro* Data

**Figure 1:**
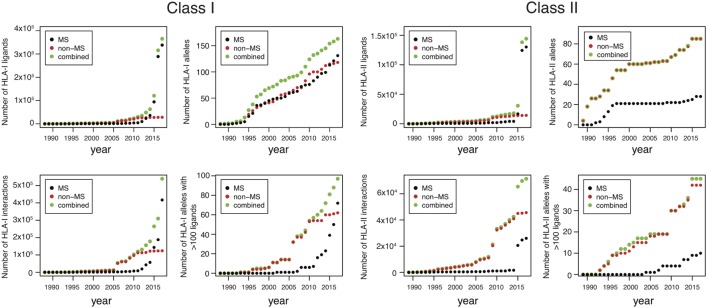
Analysis of HLA-I and HLA-II ligands obtained from human leukocyte antigen (HLA) peptidomics studies and *in vitro* assays. The number of unique HLA-I ligands, the number of unique interactions, the number of HLA alleles with at least one ligand, and the number of HLA alleles with at least 100 ligands are displayed for both class I and class II, as a function of years (cumulative distributions).

Until 2012, the number of MS datasets was significantly lower than *in vitro* data (Figure 1), which partly explains why *in vitro* binding data were mainly used for training HLA-I ligand predictors. However, the situation has changed quite dramatically over the last 4 years. Combining data from IEDB (25) together with recent HLA peptidomics studies (see Supplementary Material), we can observe that roughly 10 times more unique HLA-I ligands and three times more unique HLA-I–peptide interactions are currently available from MS studies (Figure 1, the lower number of interactions than peptides for MS data comes from the fact that several MS samples did not have HLA typing information or allele restriction could not be determined with motif deconvolution). The coverage of HLA-I alleles is also larger in HLA peptidomics samples compared to *in vitro* binding data (Figure 1). Moreover, all curves for MS data do not show signs of saturation, suggesting that these numbers are likely to further increase in the coming years, especially with the growing interest in HLA peptidomics profiling of cancer samples from patients with diverse ethnic backgrounds for neoantigen discovery (15). Similar observations hold for HLA-II ligands, where the number of unique peptides identified by MS largely exceeds the number of peptides identified in *in vitro* assays. However, the number of HLA-II alleles with documented ligands is still larger for *in vitro* binding data. This likely reflects the fact that HLA-II ligands are easier to screen in a high-throughput way using peptide microarrays, and that allele restriction in HLA-II peptidomics data is still more difficult to determine with motif deconvolution or peptide clustering than for HLA-I peptidomics data.

## Modeling HLA-I Binding Specificity

### Allele-Specific Predictors

Modeling HLA-I-binding specificity has been carried out for almost 30 years since the first evidence of HLA-I motifs. Early studies used simple sequence motifs [e.g., xLxxxxxx(L/V) for HLA-A02:01]. However, as more data started to accumulate, it became clear that simple motifs were too restrictive and not quantitative enough. To overcome these limitations, position weight matrices (PWM) (equally referred to as Position Specific Scoring Matrices or simply scoring matrices) were introduced (104–107). The basic idea is to compute the frequency of each amino acid at each position in a set of (pre-aligned) peptides. The score of a new peptide can then be computed by multiplying the PWM entries corresponding to the sequence of the new peptide (see Supplementary Material). Although the idea of computing amino acid frequencies is relatively simple to understand, several steps are important when building a predictor based on PWMs. First, one has to consider the amino acid background distribution and use this distribution to renormalize the scores (see Supplementary Material). In most existing approaches, amino acid frequencies of the human proteome have been used. However, this approach may not be fully justified when using viral epitopes to train predictors. Similarly, eluted HLA-I ligands do not show the same amino acid distribution as human proteins and much lower frequency of cysteine has been reported by ourselves and others (70, 72). As such, the optimal choice of background distribution may depend on the origin (both biological and technical) of the data. Second, in most cases, estimating the frequency of amino acids occurring only a few times (or never) at a given position is highly susceptible to statistical noise. To address this issue, pseudo-counts are often used. A widely used approach is based on the BLOSUM62 matrix (see Supplementary Material) (105, 108, 109). Third, biases due to the design of specific experiments can be found in many *in vitro* datasets. For instance, if a mutagenesis was carried out at a fairly non-specific position in a given epitope, many sequences will have identical amino acids at all positions except the one used in the mutagenesis. One way to correct for such biases is to add a weight to all peptides that is inversely proportional to the number of highly similar sequences in the dataset (see Supplementary Material).

Since the last decade, most allele-specific HLA-I ligand predictors use machine learning frameworks such as neural networks, hidden Markov Models, support vector machines, or convolutional neural networks (110–114). One attractive aspect of these models is the ability to consider potential correlations between different positions within HLA-I ligands. For instance, we recently observed in HLA-B07:02 ligands that arginine is preferred at P3 or at P6, but not at both positions at the same time (101). This type of correlation is not captured by simple PWMs. However, it is still unclear how frequent these correlations are for HLA-I ligands. In particular, although many studies reported improved predictions of HLA-I ligands using machine learning algorithms (112, 115), one has to be careful before concluding that correlation patterns are prevalent, since improvement in prediction accuracy may also result from more robust regularization frameworks. Finally, machine learning approaches are also susceptible to overfitting and correcting for potential biases in training sets can be more challenging than with simple PWMs.

### Pan-Allele Predictors

Enough experimental ligands are available for roughly 100 HLA-I alleles, which represents only a small fraction of the >12,000 HLA-I alleles observed in the human population. To address this issue, pan-allele predictors have been introduced, where the input of the algorithm consists of both the sequence of the ligand and the sequence of the HLA-I allele (or of its binding site) (107, 116–118). These algorithms are powerful at capturing correlations between amino acids in the HLA-I-binding site and in the ligand. The most widely used and likely the most elaborate pan-specific algorithm is the NetMHCpan tool (117), which includes several features specific for HLA-I molecules, such as combining peptides of different lengths in the training and incorporating peptide length preferences.

Table 1 summarizes some of the most common predictors, together with information about the algorithm that is used, the type of training data and the output.

**Table 1:** Summary of some of the most recent or most widely used human leukocyte antigen (HLA)-I predictors with available web interface or code repository.

Name	Training data	Output	Algorithm	Allele coverage	Access	Reference
NetMHC4.0	BA	BA	NN	S	http://www.cbs.dtu.dk/services/NetMHC/	(110)
NetMHCpan4.0	BA + MS	R (BA)	NN	Pan	http://www.cbs.dtu.dk/services/NetMHCpan-4.0/	(117)
MixMHCpred	MS	R	PWM	S	https://github.com/GfellerLab/MixMHCpred	(72, 120)
MHCflurry	BA	BA	NN	S	https://github.com/openvax/mhcflurry	(113)
PickPocket	BA	BA	PWM	Pan	http://www.cbs.dtu.dk/services/PickPocket/	(107)
NetMHCstabpan	BS	BS	NN	Pan	http://www.cbs.dtu.dk/services/NetMHCstabpan/	(118)
NetMHCstab	BS	BS	NN	S	http://www.cbs.dtu.dk/services/NetMHCstab/	(111)
NetMHCcons	BA	BA	C	S	http://www.cbs.dtu.dk/services/NetMHCcons/	(181)
IEDB consensus	BA	R	C	S	http://tools.iedb.org/mhci/	(182)
SMMPMBEC	BA	R	PWM	S	https://github.com/ykimbiology/smmpmbec	(104)
MHCnuggets	BA	BA	NN	S	https://github.com/KarchinLab/mhcnuggets-2.0	(183)
ConvMHC	BA	R	NN	Pan	http://jumong.kaist.ac.kr:8080/convmhc	(116)
HLA-CNN	BA	R	NN	S	https://github.com/uci-cbcl/HLA-bind	(114)
SYFPEITHI	BA + MS	R	PWM	S	http://syfpeithi.de/0-Home.htm	(106)
PSSMHCpan	BA	BA	PWM	Pan	https://github.com/BGI2016/PSSMHCpan	(184)

*Column 2, BA, binding affinity; BS, binding stability; MS, HLA peptidomics data; column 3, BA, binding affinity; R, ranking; column 4, NN, Neural network (including deep networks); PWM, position weight matrices; C, consensus; column 5, S, allele specific; Pan, pan-class I*.

### Choosing the Right Training Set

While extensive work has been performed to optimize the algorithms used in HLA-I predictors, less attention has been devoted to the choice of the training set. Prior to 2016, most approaches aimed at predicting binding affinity values (i.e., IC50) and, therefore, were trained on *in vitro* data mainly obtained from IEDB (25). Although high accuracy could be reached for many common alleles, several potential biases suggest that such data can be suboptimal for training predictors. In particular, it is important to remember that most HLA-I ligands tested *in vitro* for binding were first predicted with older versions of HLA-I ligand predictors [some exceptions that used random peptide libraries include Ref. (58)]. Unfortunately, this can induce circularity when using these data to retrain predictors, and such biases are difficult to detect and correct for. Of note, the same circularity issue can also affect several published MS datasets when HLA-I ligand predictors or motifs were used to assign allele restriction and filter noise. Here, we argue that high-quality MS data not filtered with existing predictors provide a powerful solution toward overcoming the potential circularity inherent to many *in vitro* binding data.

### Using MS Data for Identifying HLA-I Motifs and Training Predictors

Mono-allelic samples or transfected soluble HLA-I alleles have been used since many years to study the binding motifs of specific HLA-I molecules (91, 92). However, due the experimental work implied by such approaches, they were never applied to a large panel of HLA-I alleles [the largest studies consist of 16 alleles for mono-allelic cell lines (70) and 17 alleles for transfected soluble HLA-I alleles (75)]. For pooled HLA peptidomics dataset, the impossibility to experimentally assign allelic restriction was often considered as an important hurdle to use such data toward studying HLA-I-binding motifs.

However, in the last few years, it became clear that pooled HLA peptidomics data can be used to study HLA-I motifs and improve predictions, thereby overcoming the need of genetically modifying cell lines or transfecting soluble HLA-I alleles. The first attempt to determine HLA-I-binding motifs from pooled HLA peptidomics data was published in 2015 (18). A year later, we published the first evidence that such data can be used to improve predictions of HLA-I ligands (101). Since then, many studies have confirmed these results both for the identification of new motifs (72, 81, 102, 103, 119) and for improving predictions of HLA-I ligands by integrating MS data in the training of predictors (70, 72, 117, 120).

As of today, two algorithms have been used for motif deconvolution and peptide clustering of pooled HLA peptidomics data. One of them (MixMHCp) is based on mixture models and was initially developed for multiple specificity analysis in large PDZ or SH3 ligand datasets obtained by phage display (121–123). In this framework, the idea is to let the algorithm infer K distinct PWMs that optimally model the eluted peptides (101). Since peptides identified by MS come from K different HLA-I alleles (*K* ≤ 6), it is not surprising that the motifs that optimally describe the data correspond precisely to the specificity of these alleles. The other algorithm (GibbsCluster) is based on simulated annealing to group the peptides into different clusters optimizing a global cost function that models how well each peptide fits into its respective cluster (103, 124). Somehow unexpectedly, both algorithms were initially developed for other purposes (i.e., multiple specificity analysis for MixMHCp and simultaneous clustering and alignments of short peptides for GibbsCluster) and their use for motif identification in HLA peptidomics data was realized only later (18, 101, 102). The two approaches have many conceptual similarities since the likelihood function optimized in MixMHCp differs only slightly from the cost function optimized in GibbsCluster. In practice, the two algorithms lead most of the time to very similar results for HLA-I peptidomics data (101) and nearly identical motifs as those obtained from mono-allelic samples or transfected soluble alleles (72) (see also examples in Figure S1 in Supplementary Material). In some cases, as we have reported, the mixture model tends to be slightly more sensitive to identify motifs supported by few peptides, such as those describing HLA-C alleles (101). Conversely, the GibbsCluster has several advantages, such as the ability to combine peptides of different lengths and the simultaneous clustering and alignment of the peptides (which is critical for HLA-II ligands) (102, 103). Both methods can be used as command line or through webservers (see http://www.mixmhcp.org and http://www.cbs.dtu.dk/services/GibbsCluster-2.0/). The availability of these algorithms strongly supports the notion that allele assignment in MS data should not be done based on HLA-I ligand predictors, since this may remove all peptides that are not well modeled with existing predictors, and hence bias determination of motifs and prevent improving the predictors. It is also important to emphasize that accurate motif deconvolution requires a large number of peptides, and ideally, many samples to test the robustness of the motifs (72). For this reason, it is likely the combination of higher accuracy and throughput of MS instruments (18) together with these novel algorithms that enabled accurate HLA-I motifs identification in pooled HLA peptidomics data.

Annotation of the motifs deconvolved from pooled HLA peptidomics data can be done in different ways. For alleles for which a reasonable description of the motifs is known, one can simply compare the motifs found in MS data to the known references (18). Using Euclidean distance to quantify the similarity between the PWMs appears to provide stable results and most of the time the mapping is quite obvious (72, 101). If the motifs are not known, two approaches have been developed. One fully unsupervised approach was proposed by ourselves based on cooccurrence of HLA-I alleles across different samples (72). In this way, we could identify and annotate HLA-I motifs for more than 40 alleles, including 7 alleles that had no experimental ligands at the time of this study. Another semi-supervised approach that works well in most cases consists of comparing with motifs predicted from pan-allele predictors such as NetMHCpan (119).

An important limitation of motif deconvolution approaches comes from the fact that motifs for some alleles (especially HLA-C alleles) are more difficult to detect in many samples. Also, in the presence of highly similar motifs (e.g., HLA-A23:01 and HLA-A24:02, or HLA-C07:01 and HLA-C07:02), the two motifs often cannot be split (72). Because of this, not all HLA peptidomics datasets are appropriate for training predictors for each allele expressed in the corresponding sample. This limitation can be alleviated by considering large collections of HLA peptidomics studies and focusing on cases where the motifs are clearly visible and can be unambiguously annotated (72). Finally, it is sometimes useful to consider more motifs than the number of alleles in order to identify motifs for each allele (Figure S2 in Supplementary Material).

**Figure 2:**
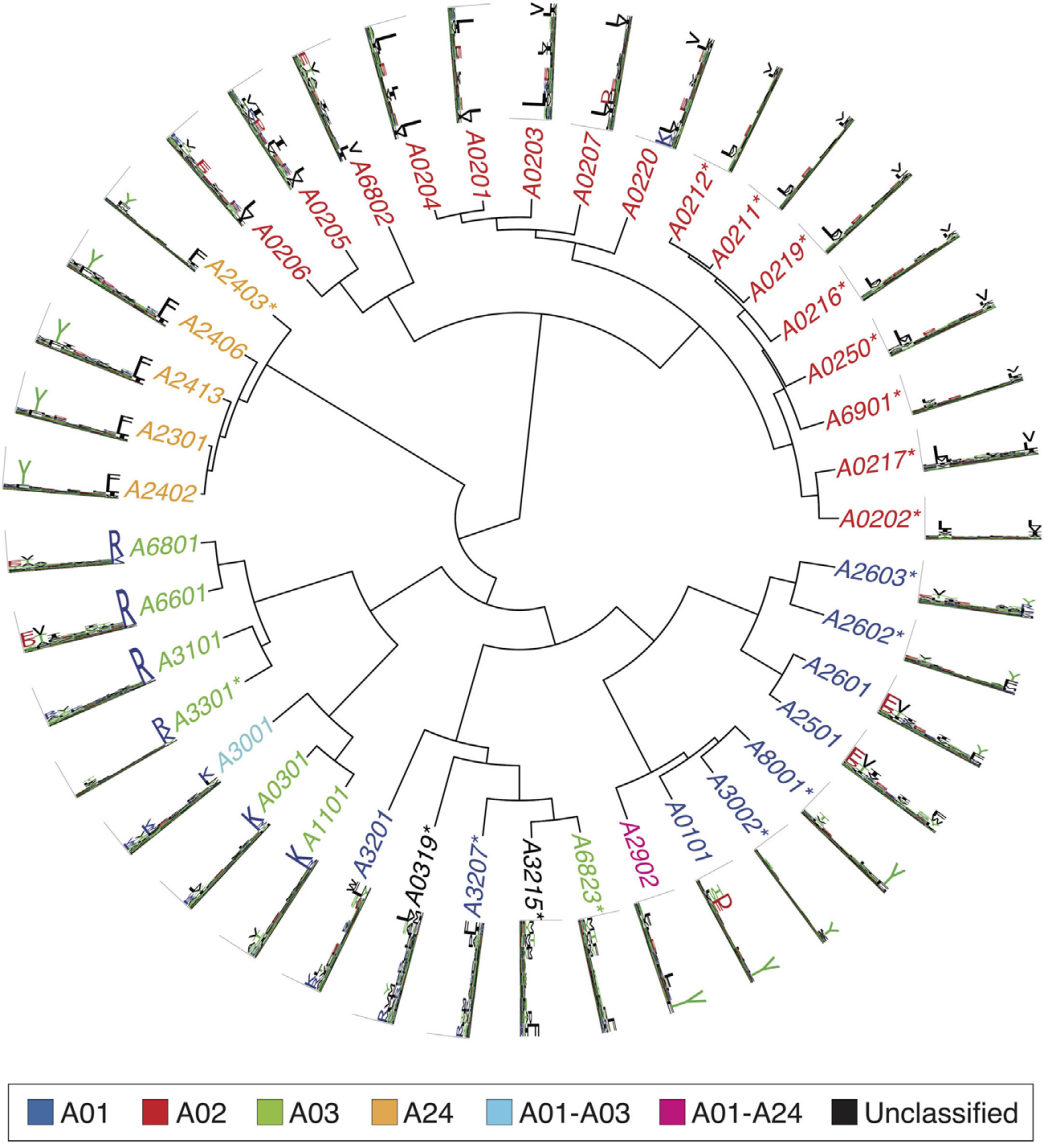
Hierarchical clustering of HLA-A alleles based on their binding specificity. Stars indicate cases where only *in vitro* binding data were available to generate the motifs. In all other cases, only mass spectrometry data were used. Name colors and their descriptions in the legend indicate supertypes as defined in Ref. (185).

**Figure 3:**
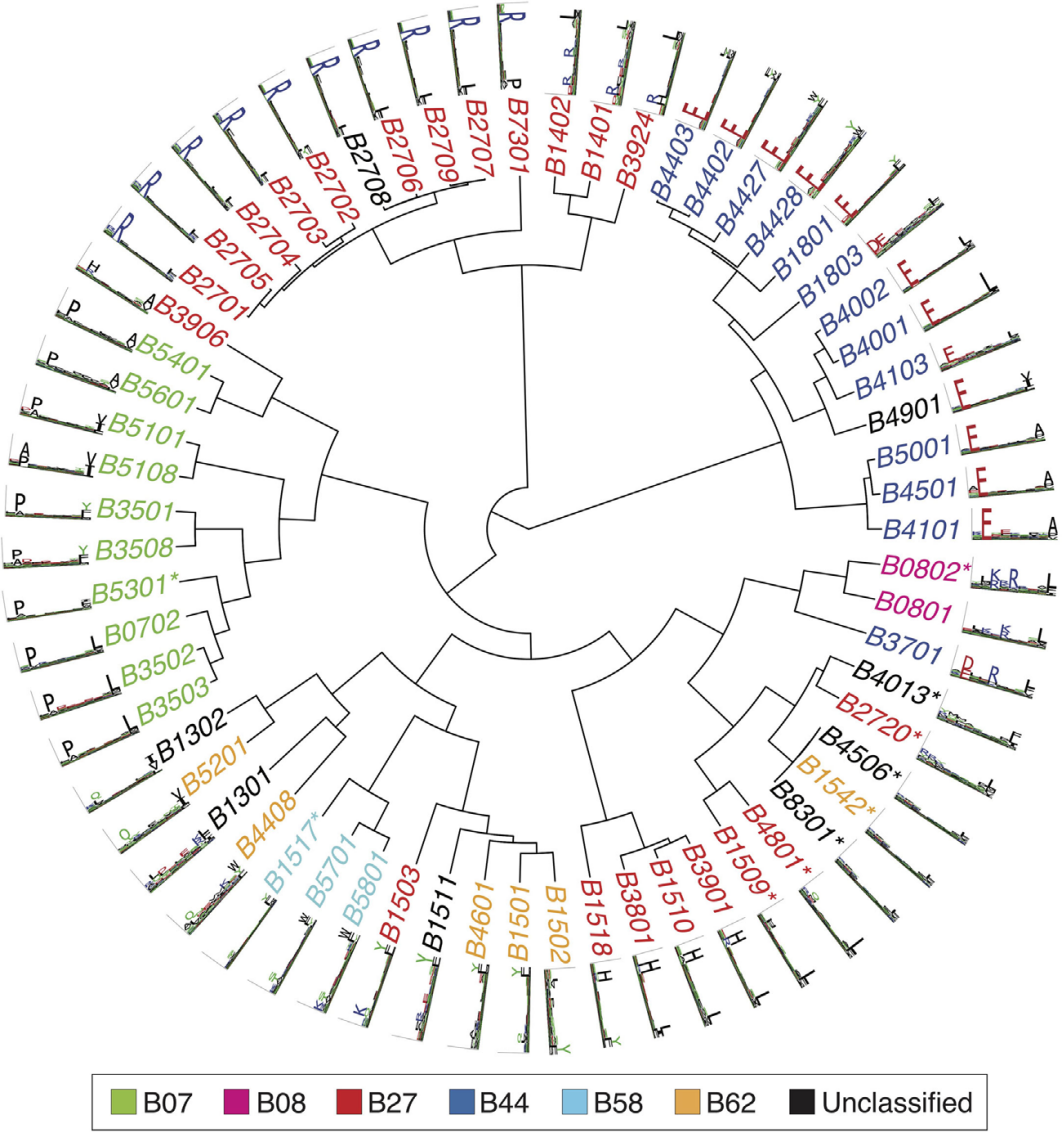
Hierarchical clustering of HLA-B alleles based on their binding specificity. Stars indicate cases where only *in vitro* binding data were available to generate the motifs. In all other cases, only mass spectrometry data were used. Name colors and their description in the legend indicate supertypes as defined in Ref. (185).

**Figure 4:**
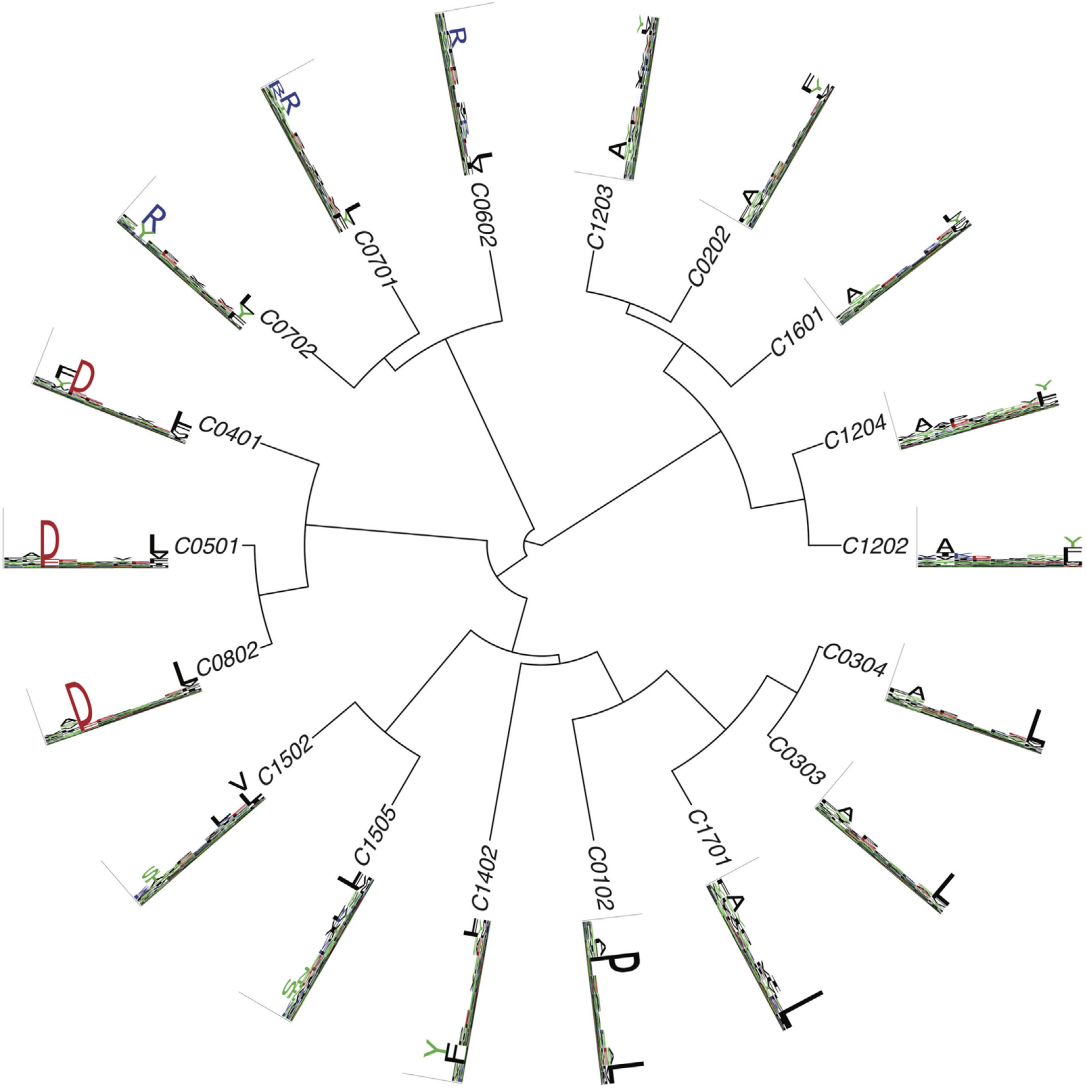
Hierarchical clustering of HLA-C alleles based on their binding specificity. Only mass spectrometry data were used. Peptides from both single allele and deconvolved pooled HLA peptidomics samples were used (see Supplementary Material).

Figures 2–4 summarize the HLA-A, HLA-B, and HLA-C motifs currently available by combining motifs deconvolved from recent MS studies together with IEDB data (see Supplementary Material). As expected, the clustering based on the similarity between the motifs (see Supplementary Material) broadly recapitulates the supertype assignment for HLA-A and HLA-B alleles and helps highlighting differences among alleles classified within the same supertypes.

### Biases in MS Data

While MS data are not suffering from the potential circularity present in many *in vitro* binding data, they are not free from any biases. First, as already mentioned, only peptides that are part of the database used for spectral searches can be detected in HLA peptidomics data, or else, the less accurate *de novo* method may be applied. This has direct implication for cysteine-containing peptides. Since this amino acid can be chemically modified by oxidation and as such modifications are typically not included in standard MS searches, cysteine occurs at very low frequency in HLA peptidomics datasets. Attempts to correct for this bias when training predictors tried to renormalize PWMs based on observed amino acid frequencies at non-anchor positions (72) or expand the MS spectral search to include modified cysteines (70). Second, peptides that are too hydrophobic or too hydrophilic might be missed applying the common purification methods that rely on retaining peptides through hydrophobic interactions with the solid phase. Furthermore, some peptides have features that make them incompatible with ionization or lead to poor fragmentation. Combining fragmentation methods, such as higher-energy collision-induced dissociation and electron-transfer dissociation, have been shown to improve spectra annotation of HLA peptides (73). Despite these limitations, inspection of HLA peptidomics data and comparison with motifs obtained from *in vitro* data did not reveal major differences, except for the low frequency of cysteine [slightly higher frequency of charged amino acids at some positions has been reported in some studies (101, 102)]. Third, immuno-purification based MS data cannot distinguish between HLA-I ligands presented on the cell surface from those resident in the ER. This can be achieved by purifying HLA-I peptides from the cell surface by mild acid elution (125, 126). However, in a head-to-head comparison, the IP method outperformed the mild acid elution in terms of peptide recovery (127). Last, when considering MS data, it is important to remember that these peptides come from human proteins and that proteins or domains within proteins can display significant homology (especially for class II ligands where in addition many peptides can originate from the same core region). This can artificially enhance the frequency of some amino acids. This issue is especially important when building random models of MS data to infer whether amino acid frequencies (either within a motif or at flanking regions) differ from what is expected by chance.

## Modeling HLA-II-Binding Specificity

Predictions of HLA-I ligands, especially with the recent incorporation of high-quality MS data in the training of predictors, have reached a high level of accuracy (70, 72, 117, 120). The situation is unfortunately not the same for HLA-II ligands, which are still much more difficult to predict despite the large amount of experimental data acquired over the years (Figure 1). Several challenges arise when modeling the binding specificity of HLA-II alleles. First, HLA-II alleles tend to have more degenerate and less specific motifs. Second, all current approaches rely on first aligning peptides with tools such as NN-align (128). Although these tools have been optimized to handle HLA-II ligands, automated alignment of small peptides is known to be a difficult computational problem. Finally, the fact that HLA-II molecules form dimers further increases the diversity for HLA-DP and HLA-DQ alleles where both members of the dimers are polymorphic. Allele-specific HLA-II ligand predictors include NetMHCII (129), ProPred Singh (130), MHCPred (131), TEPITOPE (132), and consensus methods (133). Pan-specific class II predictors mainly consist of NetMHCIIpan (129). While all these predictors show better than random performances, their accuracy is lower than for HLA-I ligand predictors. This may be due to the challenges of determining class II motifs, as well as to the complex machinery of class II presentation, whose specificity is still poorly understood from a quantitative and predictive point of view [see Ref. (7–10) for a detailed discussion of the more biological aspects of this process and the importance of HLA-DM and other enzymes]. In particular, it appears that properties such as conformational flexibility play a role in loading onto HLA-II molecules (134), and these properties are difficult to predict directly from peptide sequences.

Whether similar improvement for class II predictions as for class I will be reached by incorporating class II peptidome data in the training of algorithms has not been investigated at a large scale. Nevertheless, it has been recognized already long ago that eluted ligands could provide important information about HLA-II-binding motifs (135). More recently, HLA-II peptidomics was performed in BALB/c and C57BL/6 mice and demonstrated that clear motifs for H-2 I-Ad and H-2 I-Ab could be obtained (136). A subsequent study suggested that predictors trained on these data perform better than NetMHCIIpan when repredicting the MS data (137). A similar strategy was carried out in transgenic DR1+ and DR15+ mice to identify the motifs of these two alleles (138). Recent studies also indicate that motif deconvolution with the GibbsCluster algorithm may work in pooled HLA-II peptidome datasets (21, 139), which could lead to refinement of HLA-II motifs and improved predictions in the coming years, as suggested in a recent preprint (140). However, the results are still more challenging to interpret and some motifs predicted by GibbsCluster are difficult to annotate, while the motifs for some alleles are sometimes not detected (21, 139, 141).

## Investigating Other Properties of HLA–Peptide Interactions

Many other important properties of HLA-I molecules beyond the 9-mer-binding motifs themselves can be studied through the analysis of HLA peptidomics data.

### Peptide Length Distribution

Arguably, the most important information beyond the binding motifs that can be extracted from MS data is the characterization of peptide length distributions. Many studies have demonstrated high heterogeneity of peptide length distributions between different alleles, with alleles such as HLA-B51:01 displaying high frequency of 8-mers (only slightly smaller than 9-mers) and very few longer peptide, while others such as HLA-A01:01 show high frequency of longer (≥12 amino acids) peptides, which can still be recognized by T cells (15, 70, 97, 103, 142). Structurally, most longer peptides are known to form bulges, with anchor residues conserved at the second and last positions of the peptides. Some patterns emerged from analysis of peptide length distributions. For instance, HLA-I alleles with anchor residues at middle positions (e.g., HLA-B08:01, HLA-B14:01, HLA-B14:02, HLA-B37:01) displayed peptide length distributions peaked at 9-mers, which is consistent with the fact that the middle anchor residue needs to be structurally conserved in the presence of an anchor at such positions (101). The study by Trolle and co-authors (97) demonstrated that peptide length distributions observed in MS data for five alleles could not be simply explained by differences in binding affinity, suggesting that the pool of peptides available for loading in the ER is skewed toward 9-mers. This likely implies that predictors trained on MS data will differ from those predicting binding affinity when comparing peptides of different lengths. In a recent preprint (143), we performed a large-scale analysis of peptide length distributions across 85 HLA-I alleles and could identify clusters of HLA-I molecules based on the similarity of their peptide length distributions. Peptide length distribution has been incorporated into the latest versions of NetMHC and NetMHCpan, by adding one additional input node encoding for peptide length in the neural networks (110, 117), and into MixMHCpred by directly fitting distributions observed in MS data (143).

As observed in our recent paper (21), peptide length distribution can also be affected by different treatments such as INFγ likely due to modulating the activity of catalytic subunits of the proteasome, and these aspects are not captured by existing predictors.

### C- And N-Terminal Extensions

Human leukocyte antigen peptidomics data have been instrumental in exploring non-canonical binding modes in HLA-I ligands. In particular, several recent studies have used MS data to study C- and N-terminal extensions in HLA-I ligands. Although such extensions had been identified long ago [first crystal structure in 1994 (144), PDB:2CLR, followed by another structure in 2009 (145), PDB:3GIV], their prevalence had remained unclear. In 2016, HLA peptidomics profiling and X-ray crystallography were combined to explore C-terminal extensions in HLA-A02:01 and demonstrated that such extensions were especially common among peptides originating from pathogens (146). This was followed by additional work that better described the structural mechanisms and cellular origin of such extensions (147). N-terminal extensions have been identified in HLA-B57:01 (148) and HLA-B58:01 (149). More recently, we have demonstrated that C-terminal extension occur in a substantial fraction of HLA-I molecules and can be recognized by CD8 T cells (120). Our work further enabled us to identify both sequence and structural features predictive of such extensions. In particular, it appeared that C-terminal extensions are especially frequent in alleles displaying specificity for positively charged residues at the last anchor position (e.g., HLA-A03:01, HLA-A31:01, HLA-A68:01). While MS data potentially provide a rich source of information about C- and N-terminal extensions, identifying these extensions by looking at the sequence of the peptides can be challenging, especially when the residue at the extension has similar specificity as the anchor residue (i.e., same residues at P9 and P10 for putative C-terminal extensions, same residues a P2 and P3 for putative N-terminal extensions). Our work suggests that many ambiguous cases may actually follow the bulging conformation (120).

### Posttranslationally Modified HLA-I Ligands

Posttranslationally modified peptides have been identified by MS analysis of eluted ligands (15, 150–152). These include mainly phosphorylated peptides, which can be recognized by T cells (153–155). Phosphorylation was observed to occur mainly at position 4 (15), suggesting that it does not impact too much the binding to the HLA-I molecules. Existing HLA-I ligand predictors do not include phosphorylated peptides, although the increasingly larger MS datasets of phosphorylated HLA-I ligands suggest that predictions of phosphorylated HLAI ligands may soon become feasible. As for now, one approach is to treat the phosphorylated residue as its unmodified counterpart and use available predictors to predict such ligands.

### HLA-II Molecules

Fewer studies used MS data to investigate properties of HLA-II molecules other than the actual-binding motifs. Studies reported broad peptide length distributions peaked around 15-mers (15, 21, 139, 156, 157), but it is still unclear to what extent distinct alleles show distinct peptide length distributions. Other properties of HLA-II molecules that could be studied based on MS data include the cellular origin of class II peptides (156, 158, 159) and the impact of different biological processes such as autophagy (160). MS studies also indicated preference for proline at the second and second to last position of peptides degraded in the endolysosomal pathway (156, 161), and preference for lysine at the C-terminus and for aspartate at the N-terminally flanking residue of class II epitopes degraded in the cytosolic pathway (156). Along these lines, many studies support the idea that presentation of class II peptides is not only driven by the binding specificity to the HLA-II molecules but also involves some (still uncharacterized) specificity in the processing machinery, flanking regions (162), or presentation hotspots in the human proteome (159).

Considering the increasingly higher quality and throughput of class II HLA peptidomics data (15, 21, 86, 138, 139), we anticipate that analysis of HLA-II peptidomes will further enable researchers to investigate new properties of HLA-II molecules. For instance, it will be interesting to see whether the presence of bulging class II ligands, as recently reported from an analysis of *in vitro* binding data (163), can be confirmed in large-scale unbiased MS data.

## Antigen Presentation—Beyond Binding to HLA

### Integrating Cleavage Site and TAP Transport Predictions, Signals from Flanking Regions and Other Proteomic Information

Mass spectrometry-based HLA peptidomics analysis can reveal crucial information about the rules underlying the biogenesis of the HLA peptidome, including signatures of cleavage site specificity, influence of source protein expression or other patterns characterizing naturally presented HLA ligands. Predictions of cleavage sites have been available since many years and have been used to narrow-down the list of predicted HLA-I ligands (164). Although some improvement has been observed, cleavage site predictions have only a limited effect on prediction accuracy of naturally presented HLA ligands. For this reason, it is not widely used in many existing pipelines for neoantigen predictions from exome sequencing data, for instance. Predictions of TAP transport has also been integrated with affinity and cleavage site predictions to model antigen presentation (165–167). Interrogation of properties of thousands of HLA-I ligands source-proteins has revealed that the proteome is not randomly sampled. Several biological determinants correlate with presentation, such as level of translation (71), expression, and turnover rate (18) and selective regions of the human proteome (71). Specific amino acid signals in flanking regions of naturally presented HLA-I ligands, like lower frequency of proline, have also been demonstrated (70). While binding to HLA still appears to be the most selective step of class I antigen presentation, integrating these additional features into a single predictor further improves the accuracy of predictions of naturally presented peptides (70, 71).

### Presentation Hotspots

After deep interrogation of HLA peptidomics large scale data, we and others have recently suggested that HLA ligands are not randomly distributed along the protein sequences but are located within “hotspots” (15, 71), which fit proteasomal cleavage, peptide processing, and HLA-binding rules (168). Recently, we envisioned that these hotspots reflect regions of proteins with enhanced proteasomal or endosomal peptide production prior to HLA loading and may, therefore, provide complementary information to HLA-binding predictions (159). To this end, we collected a large dataset of MS detected HLA class I and class II ligands from different cancer and healthy tissues and variety of cell lines. We used this dataset to score potential neoantigens based on how well their un-mutated source proteins are naturally presented. In a proof of concept study, we tested this hypothesis with published data (33) and could show that MS-based features improved the prioritization of confirmed neoepitopes (159). Large scale databases of HLA peptidomics data capture the global nature of the *in vivo* peptidome averaged over many HLA alleles and, therefore, reflect the propensity of peptides to be presented, which can complement binding-affinity predictions.

## Future Perspectives

### Expanding the Description of HLA Motifs

Accurate and unbiased binding motifs are available for a bit more than 100 HLA-I alleles (Figures 1–4). This is only a tiny fraction of the >12,000 HLA-I alleles listed in IMGT/HLA database (4). For this reason, much has still to be learned about the specificity of HLA-I molecules. We anticipate that the ability to deconvolve HLA-I motifs from pooled HLA peptidomics data will play an important role to expand our understanding of HLA-I-binding specificities. This is especially promising in light of the current interest in using MS to identify neoantigens in cancer patients. However, even with the current efforts in HLA peptidomics, extrapolation of the curves in Figure 1 suggests that experimentally determined HLA-I ligands will remain available for only a small fractions of HLA-I alleles in the coming years. For this reason, development of pan-specific HLA-I ligand predictors leveraging high-quality MS data available for a few (~100) alleles to model the binding specificity of other alleles are expected to play an important role in broadening the scope of HLA-I ligand predictions to rarer alleles without document ligands (117). Accurate and in-depth HLA peptidomics data will also likely play an important role in improving our understanding and description of HLA-II motifs. Use of HLA-II gene-specific antibodies (i.e., pan-DR, pan-DP, or pan-DQ) may facilitate accurate motif deconvolution in such datasets.

### Better Understanding of Antigen Presentation

While binding to HLA molecules is the most specific and best quantitatively characterized step of the antigen presentation process, it is likely that some additional filtering comes from cleavage in the proteasome, transport with TAP, and loading in the ER. As mentioned earlier, several recent studies suggest that including these additional parameters further improves prediction accuracy (70, 71, 159, 166). One of the challenges there is to disentangle real biological signals from potential technical biases in MS data. Despite this caveat, it is likely that accumulating very large datasets of naturally presented HLA-I ligands is the only way to improve the accuracy of models of antigen presentation that go beyond the binding to HLA molecules. In addition, it could provide new information about how the HLA peptidome can be remodeled in response to extracellular signals, such as IFNγ stimulation (19, 21). We, therefore, envision that screening how inhibition or activation of components of the antigen processing and presentation affect the nature of naturally presented HLA ligands on a large scale may reveal their role in shaping the HLA peptidome.

### Non-Canonical HLA-I Ligands

Increasing evidences also suggest that non-canonical and cryptic peptides contribute to the HLA peptidome and expand the range of putative T cell epitopes. Laumont et al. have constructed a reference database of stop-to-stop translation products of six open reading frames of expressed RNAs and revealed that about 10% of the peptidome derive from presumably noncoding genomic sequences or exonic out-of-frame translation (87). Liepe et al. have reported that around 30% of the peptidome is derived from non-contiguous peptides spliced by the proteasome (169). Unexpectedly, spliced peptides displayed significantly lower predicted affinity than the normal peptides identified in the same samples (169) and did not show the expected HLA-I motifs. A very large database that is about two orders of magnitude larger than the typical protein-coding database was used to incorporate theoretical spliced products (169). Searching such large databases, especially in order to identify HLA peptides that have no enzymatic restrictions, may lead to improper control of false positives (170). In a recent preprint (171), we proposed an alternative, more conservative, approach to identify spliced peptides among HLA-I ligands based on *de novo* interpretation of high-quality spectra, suggesting that the number of such peptides may have been overestimated in the original study. The exact amount of spliced HLA-I ligands is still a matter of debate, and further studies will be needed to precisely estimate the fraction of spliced peptides actually displayed on HLA-I molecules. However, these potential issues suggest that putative spliced peptides may not all be appropriate for training HLA-I ligand predictors. Exploring non-canonical HLA ligands derived from translation of non-conventional regions in our genome or posttranslation events such as splicing is like finding a needle in a haystack. *In silico* predictions of such potential HLA ligands with existing tools may, therefore, lead to in-controlled numbers of false-positives, since the non-canonical space is theoretically orders of magnitude larger than the current canonical protein space. Hence, intensive proteogenomics based investigation of acquired HLA peptidomics data will likely play a central role in this endeavor and will require advanced computational tools and statistics to properly control for false positives.

### Toward Predictions of Immunogenicity

Recent years have witnessed an unprecedented growth of in-depth and accurate MS data (Figure 1) that significantly enhanced our ability to predict antigen presentation. Unfortunately, these data cannot inform us about the most critical step in immune recognition, namely, the recognition of presented antigens by T cells. Much less is known there, and it is for instance, a disappointing fact that most predicted neoantigens from mutations found by exome sequencing of tumors are not recognized by T cells, although many resulting peptides do bind to HLA-I molecules. While direct identification of mutated peptides presented on the surface of cancer cells will likely improve the fraction of truly immunogenic epitopes (101), it is likely that many mutated peptides seen by MS will still not be immunogenic. Moreover, although binding affinity has been demonstrated to be useful for enriching pools of peptide in immunogenic epitopes (especially for class I), many known immunogenic epitopes display low-binding affinity, suggesting that they would be missed by approaches based on affinity predictions only. This is especially true for class II epitopes, where clear evidences indicate that different enzymes, peptide exchange mediates by HLA-DM or HLA-DO, pH gradients and peptide conformational flexibility play a role in selecting immunodominant epitopes (8–10, 134). Unfortunately, currently, very little of this biological knowledge about class II antigen presentation could be used to improve predictions of class II epitopes.

Work by Calis et al. (172) suggested that some amino acids at non-anchor positions confer increased immunogenicity to HLA-I ligands. More recently, it has been observed that dissimilarity to self among mutated peptides predicted to have similar binding affinity as their wild-type counterpart can further help predicting immunogenic epitopes (173). Differences between the affinity of the wild-type and the mutated peptide, as well as stability of the MHC-I peptide interaction were also suggested to narrow down the list of immunogenic epitopes (174). Unfortunately, datasets of true immunogenic peptides from cancer or infectious diseases are still restricted to a few 100 peptides, limiting the power of machine learning approaches to infer properties of immunogenic epitopes (175, 176). This is likely the main bottleneck toward our understanding of the determinants of immunogenicity. Therefore, recent high-throughput methods for screening T cells using for instance DNA barcoded multimers have the potential to provide critical information about the differences between immunogenic and non-immunogenic peptides (46). Importantly, most of these approaches require to select *a priori* the HLA ligands to be screened [with the exception of a recent phage display system (177)]. Therefore, improved prediction of HLA ligands and antigen presentation will likely play an important role in optimizing the set of ligands currently tested for immunogenicity.

## Conclusion

The first HLA-I motifs were described almost 30 years ago by looking at sequences obtained from MS analysis of eluted MHC-I ligands (89, 90). Since then, much has been learned about HLA-I and HLA-II molecules through the analysis of their ligands. In human, this has resulted in a detailed description of HLA-I alleles binding specificities for the most common alleles and culminated with the development of pan-allele predictors. Recent years have witnessed an explosion of new high-quality data generated by MS about HLA-I ligands. Combined with advances in algorithms to analyze such data, this has led to refinement of known HLA-I motifs, discovery of new HLA-I motifs, characterization of peptide length distributions, analysis of N- and C-terminal extensions, characterization of antigen processing signals in flanking regions, analysis of the interplay between gene/protein expression, protein localization and peptide presentation, and evidences for presentation hotspots in the human proteome. For HLA-II ligands, MS studies have been recently used to study HLA-II motifs, suggesting that similar improvements may be observed there as well (21, 138–140). Moreover, the current interest in neoantigen discovery will likely result in many more HLA peptidomics datasets from donors with diverse HLA backgrounds and different pathogeneses. This will provide unique opportunities to further improve our understanding of the rules of antigen presentation. To this end, it will be crucial that raw MS data are made publicly available, and that the reporting of HLA peptidomics data will comply with the recent minimal information about an Immuno-Peptidomics Experiment (MIAIPE) guidelines (178). Databases such as IEDB (25), PRIDE (179), or the SysteMHC Atlas (180) play a key role in this process, and it is our hope that soon all journals publishing HLA peptidomics studies will require deposition of the raw MS data in PRIDE and unfiltered lists of peptides in appropriate databases, or at least accessible in supplementary datasets.

## Author Contributions

DG and MB-S designed the review and wrote the manuscript. DG analyzed the data and prepared the figures.

## Conflict of Interest Statement

The authors declare that the research was conducted in the absence of any commercial or financial relationships that could be construed as a potential conflict of interest.
