# Supplementary material for: Predicting Antigen Presentation—What Could We Learn From a Million Peptides?
Source: Front Immunol. 2018 Jul 25;9:1716. doi: 10.3389/fimmu.2018.01716 (PMC6068240; doi:10.3389/fimmu.2018.01716)
Supplement: Supplementary file 1 [file Data_Sheet_1.PDF]

# Supplementary Materials

# Predicting Antigen Presentation – what could we learn from a million peptides?

Running title: Predicting Antigen Presentation

David Gfeller[1,2,*], Michal Bassani-Sternberg[3,*]

[1] Department of Oncology, Ludwig Institute for Cancer Research, University of Lausanne, Switzerland.

[2] Swiss Institute of Bioinformatics (SIB), Lausanne, Switzerland.

[3] Department of Oncology, Ludwig Institute for Cancer Research, University Hospital of Lausanne, Switzerland.

[*]To whom correspondence should be addressed: david.gfeller@unil.ch, Michal.Bassani@chuv.ch.

# Supplementary Materials

*HLA peptidomics datasets*

Publicly available HLA-I peptidomics datasets from several recent studies in humans were considered in this work (1–12). All HLA-I peptidomics datasets were analyzed with our mixture model algorithm (MixMHCp) to identify and annotate HLA-I binding motifs, as described in our previous studies (1,13). All motifs were manually reviewed to eliminate cases where motifs of different alleles could not be separated. Larger numbers of motifs than the number of alleles were sometimes needed to identify motifs supported by few peptides (e.g., HLA-C motifs). Samples where the motifs could not be annotated were only considered in the list of peptides, but not in the list of interactions reported in Figure 1 (the same applies for samples where the HLA-I typing information was not available). For ref. (10) the raw MS data were reprocessed and were not filtered with existing predictors. For ref. (5) , the unfiltered list of peptides was kindly provided to us by the authors and motifs representing endogenous HLA-I alleles (i.e., HLA-C04:01 and HLA-B35:03) and the peptides associated to such motifs were identified with MixMHCp.

Recent HLA-II peptidomics datasets were included in the analysis of the number of class II peptides (3,14–17). Since allelic restriction was in general not known and is more difficult to predict, these data were not included in the list of HLA-II ligand interactions, only in the list of peptides in Figure 1.

*IEDB data*

IEDB data were downloaded on March 8, 2018 (18). All non-negative data were considered here (i.e., “Positive-High”, “Positive-Intermediate”, “Positive-Low” and “Positive”). MS data were identified based on “ligand presentation” identifier. All the other data (mainly binding affinity assays) were classified as *in vitro* data. IEDB data were combined with the HLA peptidomics data from the studies mentioned above and the cumulative lists of unique peptides and unique interactions are displayed in Figure 1 as a function of time (years). When computing the number of interactions, only interactions with full information about the HLA allele (e.g., HLA-A01:01) were considered.

*HLA-I ligand predictor based on Position Weight Matrices*

Here we recall a method to build PWMs describing the binding specificity of HLA-I molecules. This approach has been used in the past by different groups (19–22), including ourselves (2), but the description of the different steps is often scattered across different publications. Therefore we thought it may be useful to review the mathematical aspects of this method, which may also help understand the discussion about potential biases in HLA-I ligand datasets. Let $X = (X_1, \ldots, X_P)$ be the set of $P$ peptides of length $L$, i.e. $X_p = \left(X_p^1, \ldots, X_p^L\right)$, interacting with a given HLA-I molecule. Matrices describing the frequency of amino acid $a$ at position $l$ are computed as:

$$M_a^l = \frac{1}{P}\sum_{p=1}^{P} \delta_{a,X_p^l}$$

where $\delta_{a,b}$ stands for the standard Kronecker symbol and is equal to 1 if $a$=$b$ and 0 otherwise, $l = 1, \ldots, L$ standing for the positions along the peptides and $a = 1, \ldots, 20$, standing for the different amino acids.

*Redundant sequences*: correction for redundant sequences can be done in different ways (21). For instance, each sequence $X_p$ can be given a weight $w_p$, corresponding to the inverse of the number of sequences in X with identity with $X_p$ above a certain threshold. Similarly, sequences can be first clustered and then each sequence within a cluster receives a weight inversely proportional to the size of the cluster. The PWM is then computed as $M_a^l = \frac{1}{P^{\text{eff}}}\sum_{p=1}^{P} w_p \delta_{a,X_p^l}$, with $P^{\text{eff}} = \sum_{p=1}^{P} w_p$.

*Pseudocounts*: pseudocounts represent a way of smoothing the amino acid frequencies and are especially important for low frequency amino acids. They are equivalent to priors in Maximum Likelihood approaches (technically the exponent of a Dirichlet prior corresponds to a flat pseudocount). In its simplest form, flat pseudocounts can be used and the PWM becomes $\widehat{M}_a^l = \frac{P^{\text{eff}} M_a^l + \beta/20}{P^{\text{eff}} + \beta}$. A more powerful approach consists of using the BLOSUM62 matrix. The main idea is that if a given amino acid $b$ is observed frequently at a given position, the pseudocounts should be larger for amino acids that are similar to $b$. This idea can be quantified as $\widehat{M}_a^l = \frac{P^{\text{eff}} M_a^l + \beta \cdot g_a^l}{P^{\text{eff}} + \beta}$, with $g_a^l = \sum_{b=1}^{20} M_b^l\, q^{b \to a}$, where $q^{b \to a}$ represents the transition probability of amino acid $b$ into amino acid $a$, as given by the BLOSUM matrix (23).

*Background frequencies*: the final PWM to use as a predictor is given by $\widetilde{M}_a^l = \frac{\widehat{M}_a^l}{f_r(a)}$, with $f_r(a)$ being the background frequency of amino acid a (typically including also the pseudocount corrections, for consistency). The score of a new peptide $Y=(Y^1, \ldots Y^L)$ is then given by

$\prod_{l=1}^{L} \widetilde{M}^{l}_{Y^l}$, or alternatively as $\sum_{l=1}^{L} \log(\widetilde{M}^{l}_{Y^l})$ (pseudo-counts ensure that all entries of $\widetilde{M}$ are larger than zero).

*Clustering HLA-I motifs*

Position Weight Matrices for each HLA-I allele were built from all alleles with available ligands (9-mers only). When not enough data were available from unfiltered HLA peptidomics samples (<200 peptides), MS data from IEDB have been included. For alleles with few (<20 peptides) or no MS data available, *in vitro* binding data from IEDB have been used (stars in Figure 2 and 3). Pearson correlation coefficient between 180-dimensional vectors representing each PWM was computed for each pair of HLA-A, respectively HLA-B and HLA-C alleles. Hierarchical clustering (hclust in R, method=”ward.D2”) was applied using 1 minus the correlation coefficient as the distance between PWMs in the clustering algorithm. Supertypes were used as defined in (24).

# Supplementary Figures

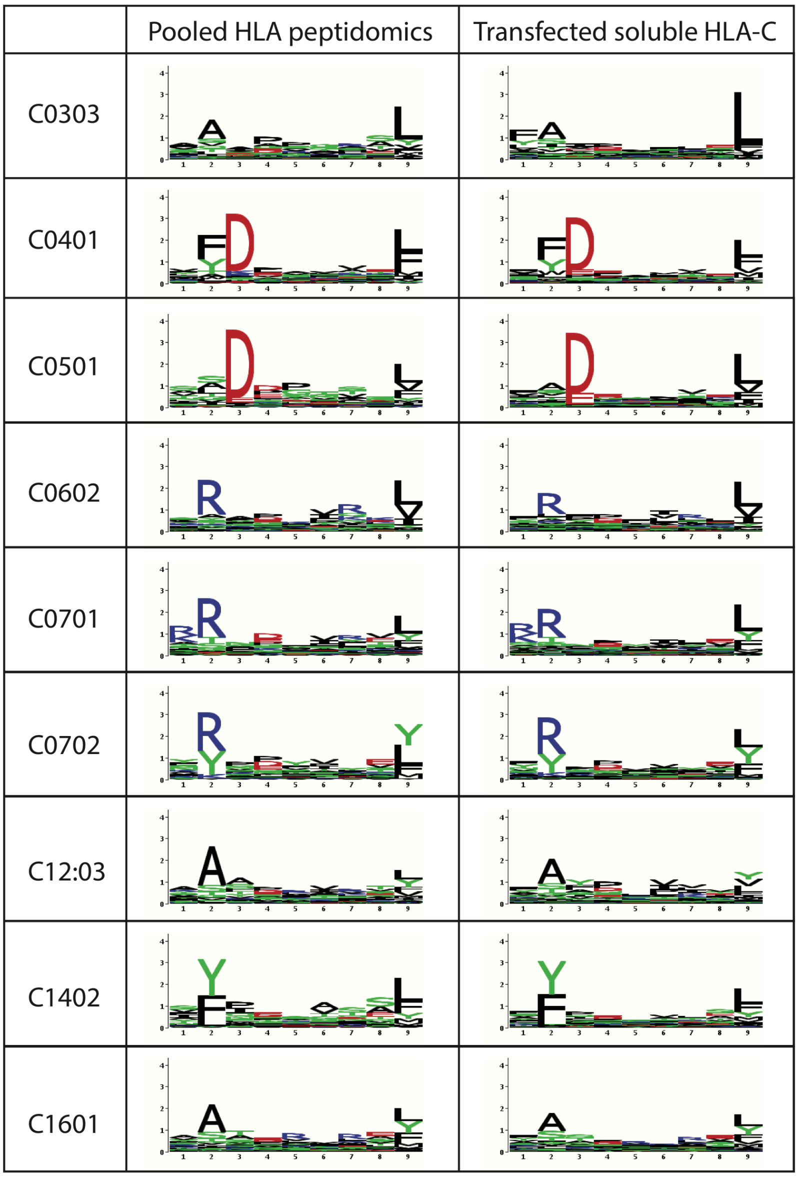


**Figure S1**: Comparison of HLA-C motifs obtained by deconvolution of pooled HLA peptidomics data (left) and HLA peptidomics profiling of cell lines with transfected soluble HLA-C alleles (right).

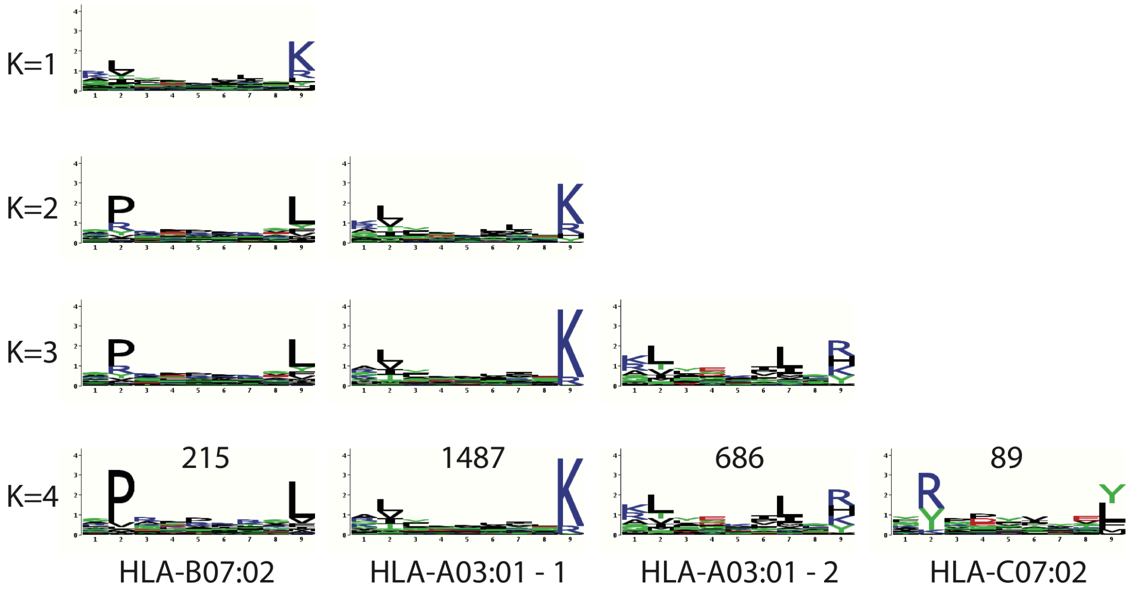


**Figure S2:** Results of the motif deconvolution with MixMHCp on HEK293 cell line HLA peptidomics data from (11). This cell line is homozygous for all HLA-I alleles (HLA-A03:01, HLA-B07:02, HLA-C07:02). Four motifs are needed to see the motif for HLA-C07:02.

# References

1. Bassani-Sternberg M, Chong C, Guillaume P, Solleder M, Pak H, Gannon PO, Kandalaft LE, Coukos G, Gfeller D. Deciphering HLA-I motifs across HLA peptidomes improves neo-antigen predictions and identifies allostery regulating HLA specificity. *PLoS Comput Biol* (2017) **13**:e1005725. doi:10.1371/journal.pcbi.1005725

2. Abelin JG, Keskin DB, Sarkizova S, Hartigan CR, Zhang W, Sidney J, Stevens J, Lane W, Zhang GL, Eisenhaure TM, et al. Mass Spectrometry Profiling of HLA-Associated Peptidomes in Mono-allelic Cells Enables More Accurate Epitope Prediction. *Immunity* (2017) **46**:315–326. doi:10.1016/j.immuni.2017.02.007

3. Bassani-Sternberg M, Bräunlein E, Klar R, Engleitner T, Sinitcyn P, Audehm S, Straub M, Weber J, Slotta-Huspenina J, Specht K, et al. Direct identification of clinically relevant neoepitopes presented on native human melanoma tissue by mass spectrometry. *Nat Commun* (2016) **7**:13404. doi:10.1038/ncomms13404

4. Bassani-Sternberg M, Pletscher-Frankild S, Jensen LJ, Mann M. Mass spectrometry of human leukocyte antigen class I peptidomes reveals strong effects of protein

abundance and turnover on antigen presentation. *Mol Cell Proteomics* (2015) **14**:658–673. doi:10.1074/mcp.M114.042812

5. Di Marco M, Schuster H, Backert L, Ghosh M, Rammensee H-G, Stevanović S. Unveiling the Peptide Motifs of HLA-C and HLA-G from Naturally Presented Peptides and Generation of Binding Prediction Matrices. *J Immunol* (2017) **199**:2639–2651. doi:10.4049/jimmunol.1700938

6. Gloger A, Ritz D, Fugmann T, Neri D. Mass spectrometric analysis of the HLA class I peptidome of melanoma cell lines as a promising tool for the identification of putative tumor-associated HLA epitopes. *Cancer Immunol Immunother* (2016) **65**:1377–1393. doi:10.1007/s00262-016-1897-3

7. Guasp P, Alvarez-Navarro C, Gomez-Molina P, Martín-Esteban A, Marcilla M, Barnea E, Admon A, López de Castro JA. The Peptidome of Behçet's Disease-Associated HLA-B*51:01 Includes Two Subpeptidomes Differentially Shaped by Endoplasmic Reticulum Aminopeptidase 1. *Arthritis & Rheumatology (Hoboken, NJ)* (2016) **68**:505–515. doi:10.1002/art.39430

8. Hilton HG, McMurtrey CP, Han AS, Djaoud Z, Guethlein LA, Blokhuis JH, Pugh JL, Goyos A, Horowitz A, Buchli R, et al. The Intergenic Recombinant HLA-B∗46:01 Has a Distinctive Peptidome that Includes KIR2DL3 Ligands. *Cell Rep* (2017) **19**:1394–1405. doi:10.1016/j.celrep.2017.04.059

9. Mommen GPM, Frese CK, Meiring HD, van Gaans-van den Brink J, de Jong APJM, van Els CACM, Heck AJR. Expanding the detectable HLA peptide repertoire using electron-transfer/higher-energy collision dissociation (EThcD). *Proceedings of the National Academy of Sciences of the United States of America* (2014) **111**:4507–4512.

10. Pearson H, Daouda T, Granados DP, Durette C, Bonneil E, Courcelles M, Rodenbrock A, Laverdure J-P, Côté C, Mader S, et al. MHC class I-associated peptides derive from selective regions of the human genome. *J Clin Invest* (2016) **126**:4690–4701. doi:10.1172/JCI88590

11. Ritz D, Gloger A, Weide B, Garbe C, Neri D, Fugmann T. High-sensitivity HLA class I peptidome analysis enables a precise definition of peptide motifs and the identification of peptides from cell lines and patients' sera. *Proteomics* (2016) **16**:1570–1580. doi:10.1002/pmic.201500445

12. Shraibman B, Kadosh DM, Barnea E, Admon A. Human Leukocyte Antigen (HLA) Peptides Derived from Tumor Antigens Induced by Inhibition of DNA Methylation for Development of Drug-facilitated Immunotherapy. *Molecular & cellular proteomics : MCP* (2016) **15**:3058–3070.

13. Bassani-Sternberg M, Gfeller D. Unsupervised HLA Peptidome Deconvolution Improves Ligand Prediction Accuracy and Predicts Cooperative Effects in Peptide-HLA Interactions. *J Immunol* (2016) **197**:2492–2499. doi:10.4049/jimmunol.1600808

14. Chong C, Marino F, Pak H, Racle J, Daniel RT, Müller M, Gfeller D, Coukos G, Bassani-Sternberg M. High-throughput and Sensitive Immunopeptidomics Platform Reveals Profound Interferonγ-Mediated Remodeling of the Human Leukocyte Antigen (HLA) Ligandome. *Molecular & cellular proteomics : MCP* (2018) **17**:533–548.

15. Khodadoust MS, Olsson N, Wagar LE, Haabeth OAW, Chen B, Swaminathan K, Rawson K, Liu CL, Steiner D, Lund P, et al. Antigen presentation profiling reveals recognition of lymphoma immunoglobulin neoantigens. *Nature* (2017) **543**:723–727.

16. Ritz D, Sani E, Debiec H, Ronco P, Neri D, Fugmann T. Membranal and Blood-Soluble HLA Class II Peptidome Analyses Using Data-Dependent and Independent Acquisition. *Proteomics* (2018) **34**:1700246.

17. Mommen GPM, Marino F, Meiring HD, Poelen MCM, van Gaans-van den Brink JAM, Mohammed S, Heck AJR, van Els CACM. Sampling From the Proteome to the Human Leukocyte Antigen-DR (HLA-DR) Ligandome Proceeds Via High Specificity. *Molecular & cellular proteomics : MCP* (2016) **15**:1412–1423.

18. Vita R, Overton JA, Greenbaum JA, Ponomarenko J, Clark JD, Cantrell JR, Wheeler DK, Gabbard JL, Hix D, Sette A, et al. The immune epitope database (IEDB) 3.0. *Nucleic Acids Res* (2015) **43**:D405-412. doi:10.1093/nar/gku938

19. Andreatta M, Lund O, Nielsen M. Simultaneous alignment and clustering of peptide data using a Gibbs sampling approach. *Bioinformatics (Oxford, England)* (2013) **29**:8–14.

20. Krejci A, Hupp TR, Lexa M, Vojtesek B, Muller P. Hammock: a hidden Markov model-based peptide clustering algorithm to identify protein-interaction consensus motifs in large datasets. *Bioinformatics (Oxford, England)* (2016) **32**:9–16.

21. Lund O, Nielsen M, Lundegaard C, Keşmir C, Brunak S. *Immunological Bioinformatics*. MIT Press. (2005).

22. Nielsen M, Lundegaard C, Worning P, Hvid CS, Lamberth K, Buus S, Brunak S, Lund O. Improved prediction of MHC class I and class II epitopes using a novel Gibbs sampling approach. *Bioinformatics (Oxford, England)* (2004) **20**:1388–1397.

23. Henikoff S, Henikoff JG. Amino acid substitution matrices from protein blocks. *Proceedings of the National Academy of Sciences of the United States of America* (1992) **89**:10915–10919.

24. Sidney J, Peters B, Frahm N, Brander C, Sette A. HLA class I supertypes: a revised and updated classification. *BMC immunology* (2008) **9**:1.
